# Exogenous Melatonin Enhances Photosynthetic Capacity and Related Gene Expression in A Dose-Dependent Manner in the Tea Plant (*Camellia sinensis* (L.) Kuntze)

**DOI:** 10.3390/ijms23126694

**Published:** 2022-06-15

**Authors:** Ni Yang, Miao-Hua Han, Rui-Min Teng, Ya-Zhuo Yang, Ya-Hui Wang, Ai-Sheng Xiong, Jing Zhuang

**Affiliations:** 1 Tea Science Research Institute, College of Horticulture, Nanjing Agricultural University, Nanjing 210095, China; 2021204040@stu.njau.edu.cn (N.Y.); 2018104089@njau.edu.cn (M.-H.H.); 2018204036@njau.edu.cn (R.-M.T.); 2018104090@njau.edu.cn (Y.-Z.Y.); 2 State Key Laboratory of Crop Genetics and Germplasm Enhancement, Nanjing Agricultural University, Nanjing 210095, China; 2019204036@njau.edu.cn (Y.-H.W.); xiongaisheng@njau.edu.cn (A.-S.X.)

**Keywords:** *Camellia sinensis*, melatonin, chlorophyll, photosynthetic characteristics, gene expression

## Abstract

The enhancement of photosynthesis of tea leaves can increase tea yield. In order to explore the regulation mechanism of exogenous melatonin (MT) on the photosynthetic characteristics of tea plants, tea variety ‘Zhongcha 108’ was used as the experimental material in this study. The effects of different concentrations (0, 0.2, 0.3, 0.4 mM) of melatonin on the chlorophyll (Chl) content, stomatal opening, photosynthetic and fluorescence parameters, antioxidant enzyme activity, and related gene expression of tea plants were detected and analyzed. The results showed that under 0.2-mM MT treatment, chlorophyll (Chl) content, photosynthetic rate (*P*_n_), stomatal conductance (*G*_s_), intercellular CO_2_ concentration (*C*_i_), and transpiration rate (*T*_r_) improved, accompanied by a decrease in stomata density and increase in stomata area. Zero point two millimolar MT increased Chl fluorescence level and superoxide dismutase (SOD) activity, and reduced hydrogen peroxide (H_2_O_2_) and malondialdehyde (MDA) contents, indicating that MT alleviated PSII inhibition and improved photochemical efficiency. At the same time, 0.2 mM MT induced the expression of genes involved in photosynthesis and chlorophyll metabolism to varying degrees. The study demonstrated that MT can effectively enhance the photosynthetic capacity of tea plants in a dose-dependent manner. These results may promote a comprehensive understanding of the potential regulatory mechanism of exogenous MT on photosynthesis in tea plants.

## 1. Introduction

The tea plant (*Camellia sinensis* (L.) O. Kuntze) is known as a leaf-type beverage crop [1]. Tender buds and leaves of the tea are usually picked to produce tea. Tea beverages have significant health benefits for humans with a unique flavor due to the existence of characteristic secondary metabolites, such as theanine, tea polyphenols, caffeine, and ascorbic acid, and are, therefore, popular all over the world [2,3,4]. Photosynthesis provides substrate and energy for the biosynthesis of secondary metabolites which are obtained from primary metabolites [5]. As the main organ for tea plant growth and development, the tea leaf is the basic site for plants to obtain energy. It fixes carbon through photosynthesis and accumulates nutrients. Light affects the metabolism of tea plants, and then affects the yield and quality of tea. Melatonin (N-acetyl-5-methoxy-tryptamine, MT) is involved in all stages of plant growth and development and can be used as a dark signal to regulate the circadian and photoperiod [6]. However, the regulation of melatonin on photosynthesis in tea plants remains unclear.

Photosynthesis serves as one of the major determinants of carbon balance and growth in plants, and is a complex process that converts light energy into chemical energy [7]. The photosynthates can be used as the carbon skeleton for plant assembly, or provide power for cell metabolism and material transport through respiration [8]. The ability and efficiency of photosynthesis are the bottlenecks to improve productivity. Carbon assimilation is integrated into the whole growing season and crop canopy. Therefore, if other constraints are not restricted, a small increase in net photosynthetic rate (*P*_n_) can be converted into substantial increase in yield [9]. The improvement in photosynthetic efficiency of tea plants can increase tea yield.

Melatonin, a multifunctional signaling molecule, was initially discovered in the bovine pineal tissue in 1958 and identified in plants in 1995 [10]. It can influence flowering of short-day plants, such as affecting photoperiodic flower induction or flower development in the early stages of Chenopodium rubrum [11]. MT significantly promoted soybean growth and fatty acid content through the enhancement of photosynthesis and sugar metabolism [12]. Additionally, the important role of MT in living organisms is to scavenge reactive oxygen species (ROS), reactive nitrogen species (RNS), and lipid radicals, as well as improve antioxidant activities, prevent cells, tissues, and organisms from oxidative stress [12,13]. MT-rich transgenic rice plants exhibited higher superoxide dismutase (SOD) and catalase (CAT) activities and herbicide resistance compared with transgenic control [14]. Compared with high concentrations (300, 500 µM), a relatively low concentration (100 µM) of MT treatment promoted the germination rate and germination index of Naked Oat seedlings [15]. Low concentrations of MT (10~30 µM) promoted the growth of primary roots and fresh weight of the seedlings of *Arabidopsis thaliana*, while high doses of MT (200~400 µM) had a poor promotion effect, and even had harmful effects in some cases [16]. MT serves as a multifunctional factor in the plant kingdom and is widely involved in stress response, growth, and development regulation of plants. 

Wang and Zhang’s groups have confirmed the positive regulation of MT on photosynthetic efficiency [17,18]. Metabolomics analysis revealed that MT regulated metabolic processes, including chlorophyll synthesis, carbon fixation, and sugar metabolism, of grafted Carya cathayensis under drought stress [19]. The potential role of MT in protecting photosynthetic apparatus was found by monitoring maximum photochemical quantum yield of PSII (*F*v/*F*m) in apple leaves [20]. Exogenous MT increased the number of open reaction centers of photosystem II (PSII), thus improving the photochemical quantum yield of PSII in Chara australis [21]. Information about the role of MT in the photosystem in tea plants remains limited, and the specific process needs further study. Research on the potential interaction between MT and cell photosynthetic reactions are rarely reported in tea plants.

The present study aimed to investigate the effect of exogenous MT treatment on photosynthesis of tea plants. We detected changes in physiology and molecules in response to different concentrations of MT at days 0, 8, and 16, respectively. Chl content, photosynthetic parameters, including photosynthetic rate (*P*_n_), stomatal conductance (*G*_s_), intercellular CO_2_ concentration (*C*_i_), and transpiration rate (*T*_r_), were measured to explore the effects of MT on tea plants. Stomatal characteristics and Chl fluorescence parameters were also detected. The antioxidant properties that MT induced in tea plants were determined by measuring the activities of antioxidant enzymes and hydrogen peroxide (H_2_O_2_) and malondialdehyde (MDA) contents. From the analysis of chlorophyll content, stomatal opening, photosynthetic and fluorescence parameters, treatment with 0.2 mM MT showed the most effect among the three treatments. Then, the tea plant with a 0.2-mM MT treatment was used in further transcriptome analysis. Moreover, the genes involved in photosynthesis and the Chl metabolic pathway were identified based on transcriptome data, and the expression gene profiles were detected by RT-qPCR. This study provides potential references to unraveling the response of MT on photosynthesis of tea plant.

## 2. Results

### 2.1. Effects of Exogenous MT Application on the Contents of Chl in Tea Leaves

The contents of Chl a, b, and total Chl with different concentrations of MT treatments are shown in Table 1. Chl a and b contents in the control group were reduced at days 8 and 16, resulting in a decrease in total Chl content. Compared to day 0, MT treatment at 0.2 mM continuously increased the total Chl content, which was 33.92% higher at day 16 than day 0. The Chl content of tea leaves under a 0.3-mM MT treatment declined at day 8, and then increased at day 16. The reduction in Chl content at day 8 was consistent with the control group, indicating that MT had no significant effect on Chl content at that time. The total Chl content under a 0.4-mM MT treatment decreased at day 8, and the reduction was lower than that of the control. Our results suggested that MT application could increase the Chl content or delay Chl degradation of tea leaves. 

### 2.2. Effects of Exogenous MT Application on Photosynthetic Parameters in Tea Plants

**Figure 1:**
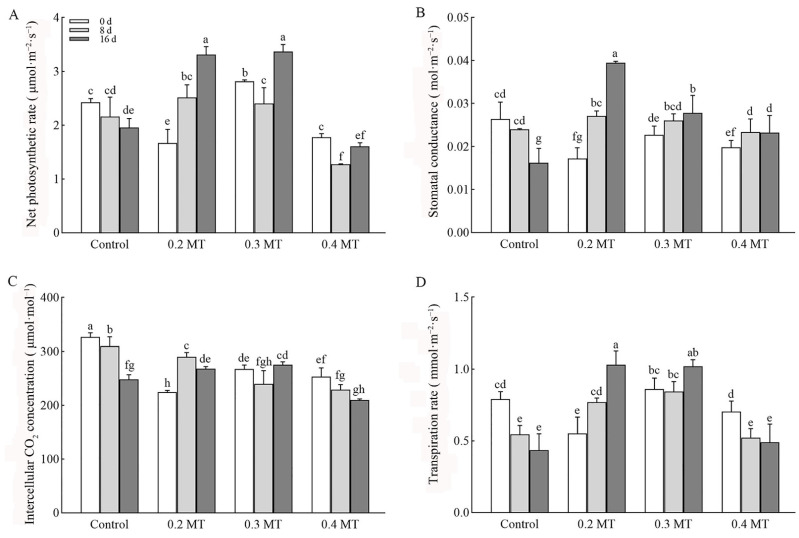
Effect of exogenous MT on *P*_n_ (**A**), *G*_s_ (**B**), *C*_i_ (**C**), and *T*_r_ (**D**). The tea plants were exposed to control, 0.2 mM melatonin (0.2 MT), 0.3 mM melatonin (0.3 MT), and 0.4 mM melatonin (0.4 MT). Data are shown as means ± SD in three independent replicates. Different superscript letters indicate significant differences between different treatments at *p* < 0.05 level.

The effects of MT on photosynthesis are presented in Figure 1. The net photosynthetic rate (*P*_n_), stomatal conductance (*G*_s_), intercellular CO_2_ concentration (*C*_i_), and transpiration rate (*T*_r_) decreased in the control group during the treatment times, especially at day 16. Exogenous MT improved these parameters. Compared to day 0, tea leaves under a 0.2-mM MT treatment had higher *P*_n_, *G*_s_, *C*_i_, *T*_r_ levels at days 8 and 16. The *P*_n_ and *G*_s_ of tea leaves treated with 0.3 mM MT at day 16 were higher than at day 0, without obvious changes in *C*_i_ and *T*_r_. MT treatment at 0.4 mM had a negative effect on *P*_n_ and *C*_i_, the decrease in them was higher than that of the control at day 8. The above data indicated that the application of 0.2 and 0.3 mM MT improved photosynthesis of tea leaves.

### 2.3. Effects of Exogenous MT Application on Stomatal Characteristics in Tea Leaves

**Figure 2:**
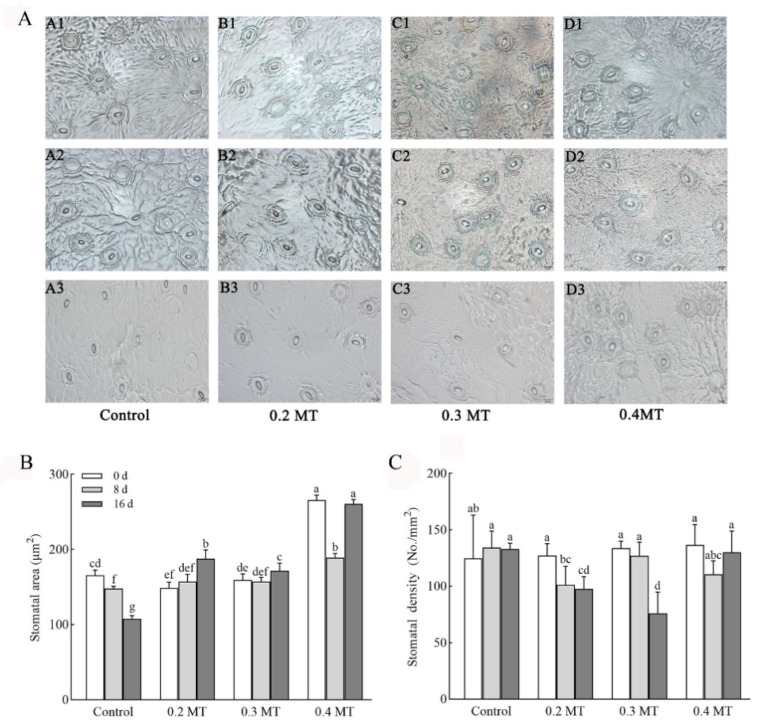
Effect of exogenous MT on stomatal morphology (**A**) stomatal area (**B**), and density (**C**). A, B, C, and D represent control group, 0.2 mM MT, 0.3 mM MT, and 0.4 mM MT treatment groups, respectively. A1, A2, and A3 represent control treatment at days 0, 8, and 16, respectively. The rest can be done in the same manner. Scale bars = 20 μm. Data are shown as means ± SD in three independent replicates. Different superscript letters indicate significant differences between different treatments at *p* < 0.05 level.

We observed the stomatal morphology (Figure 2A) under different concentrations of MT treatments and calculated the stomatal area (Figure 2B) and density (Figure 2C). Compared to day 0, the stomatal area of the control reduced significantly over time, and decreased by 35.02% at day 16 (Figure 2B). The stomatal area increased under 0.2- and 0.3-mM MT treatments at day 16, which was 1.26 and 1.08 times that at day 0, respectively. At day 8, a 0.4 mM MT treatment accelerated the decrease in stomatal area, but it showed the opposite trend at day 16. There was no significant change in stomatal density of the control (Figure 2C). Leaves exposed to 0.2 and 0.3 mM MT had lower stomatal density than that at day 0, which decreased by 23.40 and 43.24% at day 16, respectively. The trend of stomatal density under a 0.4-mM MT treatment was consistent with that of the control.

### 2.4. Effects of Exogenous MT Application on Chl Fluorescence Parameters in Tea Plants

#### 2.4.1. Effects of Exogenous MT Application on *F*v/*F*m in Tea Plants

The Chl fluorescence parameter *F*v/*F*m is often used to determine the photosynthetic physiological state of leaves. The effects of exogenous MT application on *F*v/*F*m of tea leaves are listed in Table 2. In the control group, compared with day 0, *F*v/*F*m had no significant difference on day 8 and decreased on day 16. In the 0.2-mM MT treatment group, *F*v/*F*m showed a significant upward trend compared with day 0, reaching the maximum (0.793) at day 16. The decrease in *F*v/*F*m in tea leaves treated with 0.3 mM MT was higher than that in the control group at day 16, which indicated that MT treatment with this concentration displayed an inhibitory effect on *F*v/*F*m. Compared with day 0, the 0.4-mM MT treatment increased *Fo* and significantly reduced *F*v/*F*m in tea leaves at days 8 and 16. Therefore, high concentrations of MT (0.3 mM and 0.4 mM) had negative effects on *F*v/*F*m. 

#### 2.4.2. Effects of Exogenous MT Application on Chl Fluorescence Quenching Coefficients

**Figure 3:**
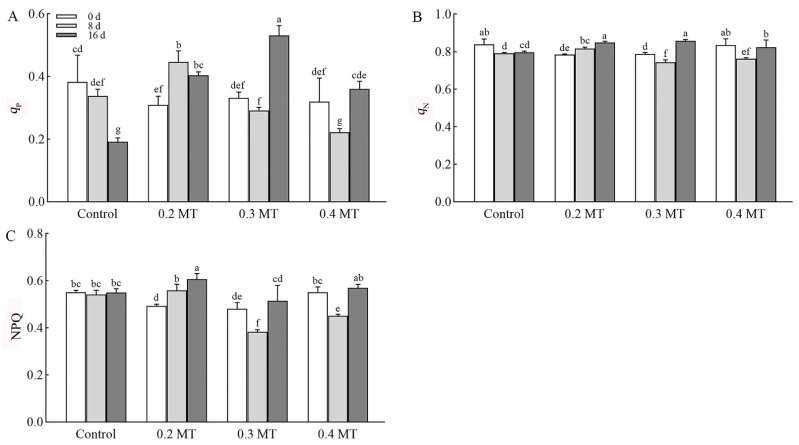
Effect of exogenous MT on Chl fluorescence quenching coefficients. (**A**) Photochemical quenching coefficient (*q*_P_): the share of light energy absorbed by PSII antenna pigment used for photochemical electron transfer, reflecting the level of plant photosynthesis. (**B**) Non-photochemical quenching coefficient (*q*_N_) and (**C**) Non-photochemical quenching (NPQ): the thermal dissipation of excitation energy by PSII antenna pigment system, reflecting the thermal dissipation level of non-photochemical process. The difference between the two is that NPQ characterizes *q*_N_, and different calculation methods. Data are shown as means ± SD in three independent replicates. Different superscript letters indicate significant differences between different treatments at *p* < 0.05 level.

The variable Chl fluorescence fraction quenched by photochemical or non-photochemical process is represented by *q*_P_ and *q*_N_, respectively. Photochemical quenching reflects the redox state of PSII primary quinone electron acceptor (QA), and high *q*_P_ level indicates strong electron transfer activity of PSII. In the control group, the *q*_P_ decreased significantly at day 16 (Figure 3A). Under 0.2-mM MT treatment, the level of *q*_P_ increased significantly at days 8 and 16, which was 1.44 and 1.31 times of that before treatment, respectively. MT treatment at 0.3 mM had a promoting effect on *q*_P_ at day 16, for which the level was increased by 3.14%. The *q*_P_ level was decreased at day 8 and then increased at day 16 under 0.4-mM MT treatment. Non-photochemical quenching mechanisms can dissipate excessive light energy absorbed by leaves in the form of heat. The *q*_N_ level of tea leaves without MT treatment at days 8 and 16 were significantly lower than that at day 0 (Figure 3B). Under 0.2-mM MT treatment, the *q*_N_ level gradually increased during the treatment times, which was 1.04 and 1.08 times at days 8 and 16 of that before treatment, respectively. The trend of *q*_N_ under a 0.3-mM MT treatment showed a pattern similar to that of 0.4 mM MT, which decreased at day 8 and then increased at day 16. The NPQ value reflects the heat dissipation level of non-photochemical process. The effects of MT treatments with various concentrations on the level of NPQ showed a high similarity with *q*_N_ (Figure 3C). Tea leaves exposed to 0.2 mM MT had the highest NPQ level at day 16.

#### 2.4.3. Effects of Exogenous MT Application on the Light Response Curve in Tea Plants

**Figure 4:**
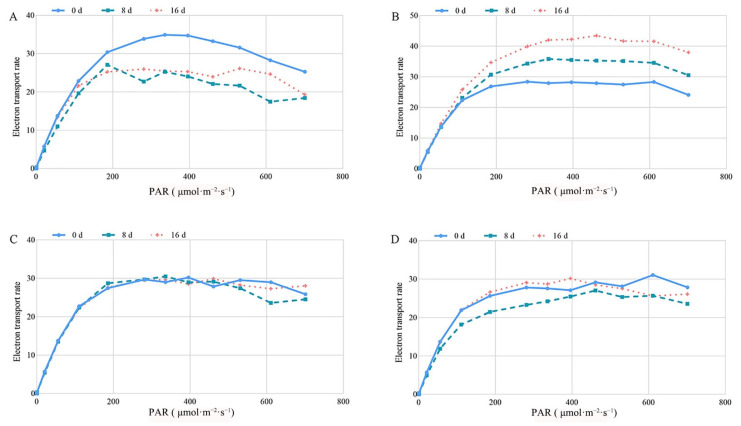
Effect of exogenous MT on ETR. The tea plants were exposed to control (**A**), 0.2 mM MT (**B**), 0.3 mM MT (**C**), and 0.4 mM MT (**D**).

By fitting the light response curve, the effect of MT on electron transfer rate (ETR) of tea leaves under different photosynthetically active radiance (PAR) was investigated (Figure 4). The ETR in each group improved with the increase in PAR, and then the growth rate slowed until it decreased. Compared to day 0, the electron transfer ability of the control decreased at days 8 and 16, while gradually increasing over time under a 0.2-mM MT treatment (Figure 4A,B). Additionally, the electron transport capacity remained relatively stable of tea leaves treated with 0.3 mM and 0.4 mM MT in comparison with control group (Figure 4C,D). Our results indicated that MT improved or maintained the photosynthetic capacity of tea plants.

### 2.5. Effects of Exogenous MT Application on H_2_O_2_ and MDA Contents in Tea Plants

**Figure 5:**
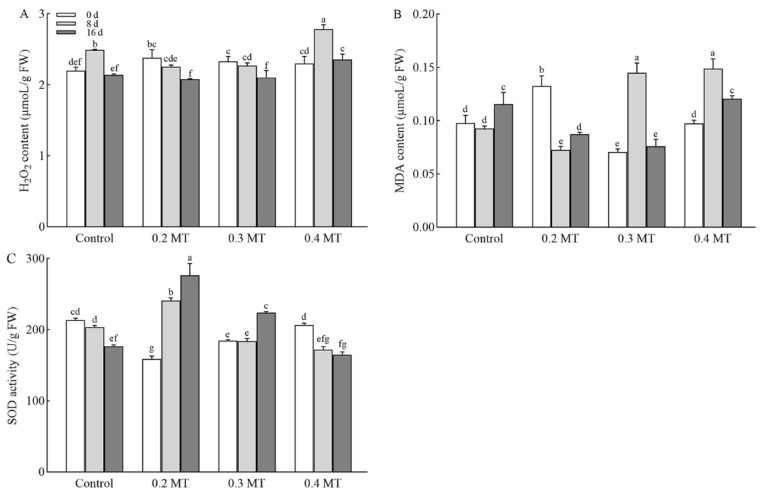
Effect of applied MT on H_2_O_2_ (**A**) and MDA (**B**) contents and SOD (**C**) activity. Data are shown as means ± SD in three independent replicates. Different superscript letters indicate significant differences between different treatments at *p* < 0.05 level.

The change in H_2_O_2_ content in MT treatments is shown in Figure 5A. In the control group, the content of H_2_O_2_ at day 8 was 1.13 times that at day 0. Treatment with 0.2 mM MT reduced H_2_O_2_ content by 12.70 % at day 16 in comparison with day 0, and with a 0.3-mM MT treatment, it decreased by 9.72%. The trend of H_2_O_2_ content under 0.4-mM MT treatment was consistent with that of the control, and the increase was higher than that of the control at day 8. The MDA content is evaluated in tea leaves at different MT concentrations and was used to assess the degree of membrane damage. The accumulation of MDA was obviously suppressed in the leaves treated with 0.2 mM MT, especially at day 8 (Figure 5B). Under the 0.3-mM MT treatment, MDA content significantly increased and then declined over time. At day 16, the increase was suppressed in comparison with the control. The MDA content of leaves treated with 0.4 mM MT increased by 34.57% at day 8, and the same increase was found at day 16 as that of the control. This result showed that MT might inhibit ROS accumulation and alleviate the oxidation of membrane lipids. The addition of 0.2 mM MT showed the greatest inhibitory effect, while 0.4 mM MT had the opposite effect.

### 2.6. SOD Activity as Influenced by Exogenous MT Application in Tea Leaves

In order to explore the effect of exogenous MT on antioxidant enzyme activity, we monitored the activities of SOD (Figure 5C). In the control group, SOD activity decreased at day 16. MT treatment at 0.2 mM increased SOD activity at days 8 and 16 in comparison with day 0, which was 1.52 and 1.74 times that at day 0, respectively. Leaves treated with 0.3 mM MT had higher SOD activity at day 16 than that day 0. Under the 0.4-mM MT treatment, the activity of SOD was decreased over time, and the reduction was higher than that of the control at day 8.

### 2.7. Transcriptome-Based Identification of DEGs Involved in Photosynthesis under MT Treatments

**Figure 6:**
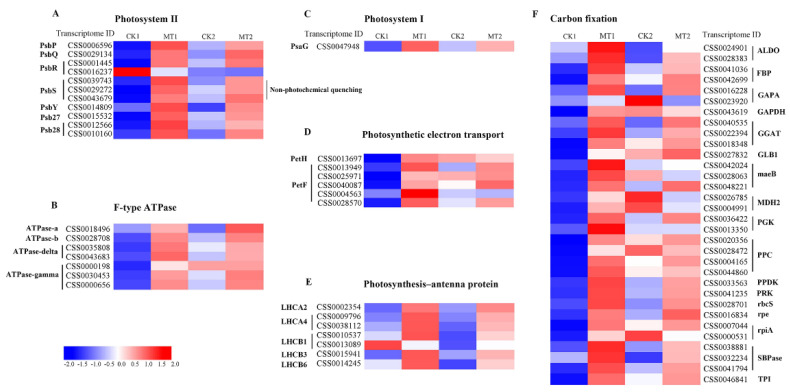
Heatmap of expression levels of photosynthesis related genes identified in DEGs. Blue and red represent low and high expression, respectively. (**A**) Photosystem II; (**B**) F-type ATPase; (**C**) Photosystem I; (**D**) Photosynthetic electron transport; (**E**) Photosynthesis-antenna protein; (**F**) Carbon fixation.

The genes related to photosynthesis were screened from the tea transcriptome, and their sequences were compared with the tea reference genome to further verify their molecular functions. CK1 and MT1 represent the control and 0.2-mM treatment groups at day 8, respectively, while CK2 and MT2 represent day 16. Among them, a total of 64 DEGs were identified in photosystem (Figure 6A–E) and carbon fixation (Figure 6F). Genes involved in PSII (*PsbP*, *PsbQ*, *PsbR*, *PsbS*, *PsbY*, *Psb27*, and *Psb28*) and photosystem I (PSI) (*PsaG*) core proteins showed upregulation in MT1 and MT2 in comparison with the control group, except *PsbR* (*CSS0016237*) (Figure 6A,C). Among them, three *PsbS* genes (*CSS0039743*, *CSS0029272*, and *CSS0043679*) were associated with non-photochemical quenching. The photoreactions include absorption of light energy by photosynthetic pigments, photosynthetic electron transfer (*PetH* and *PetF*), and ATP synthesis (ATPase-a, ATPase-b, ATPase-delta, and ATPase-gamma). Sixty-two and fifty-four DEGs encoding these proteins showed upregulation in MT1 and MT2 relative to that CK1 and CK2, respectively (Figure 6B,D). Six genes of light-harvesting complex I and II Chl a/b binding proteins that acted as photosynthesis antenna systems, *LHCA2* (*CSS0002354*), *LHCA4* (*CSS0009796* and *CSS0038112*), *LHCB1* (*CSS0010537*), *LHCB3* (*CSS0015941*), and *LHCB6* (*CSS0014245*) were upregulated under MT treatment (Figure 6E). In the carbon fixation pathway, compared with the corresponding control group, all the identified DEGs were found to be upregulated in MT1, while seven genes had the opposite expression in MT2 (Figure 6F). 

### 2.8. Transcriptome-Based Identification of DEGs Involved in Chl Metabolism under MT Treatments

**Figure 7:**
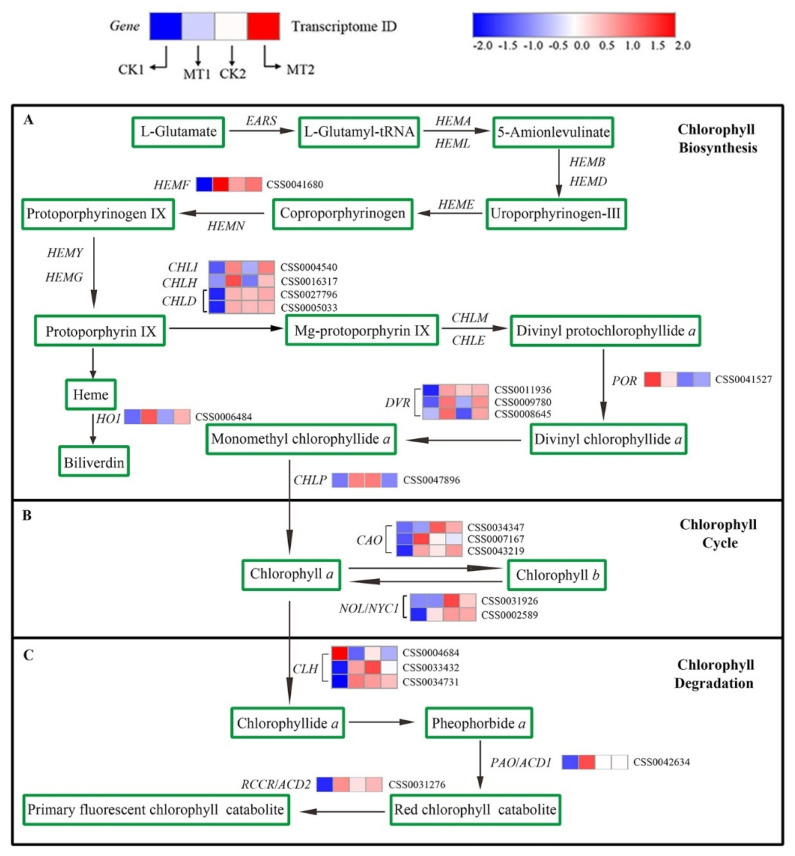
Chl metabolic pathway and heatmap of expression levels of Chl metabolism related genes identified in DEGs. (**A**) Chlorophyll Biosynthesis; (**B**) Chlorophyll Cycle; (**C**) Chlorophyll Degradation. Blue and red represent low and high expression, respectively. The different vertical columns represent different treatments, from left to right: CK1, MT1, CK2, and MT2.

The pathway of Chl metabolism is shown in Figure 7, and a total of 21 DGEs identified from the transcriptome were involved in Chl metabolism under MT treatments. Among the DEGs related to Chl biosynthesis and the cycle pathway, a total of 15 and 14 genes had higher expression levels in MT1 and MT2 treatments than that of the CK1 and CK2, respectively (Figure 7A,B). In addition, among the five DEGs involved in Chl degradation pathway, one and three genes were downregulated in MT1 and MT2 relative to that CK1 and CK2, respectively (Figure 7C). Based on RNA-seq, 3 three genes identified with CLH, which regulate Chl degradation, were downregulated under MT treatment at day 16 in comparison with CK2. Expression profiles showed that MT regulated the transcription level of genes which participate in Chl metabolic processes.

### 2.9. Expression Profiles of Genes Involved in Photosynthesis and Chl Metabolism in Tea Leaves under MT Treatments

**Figure 8:**
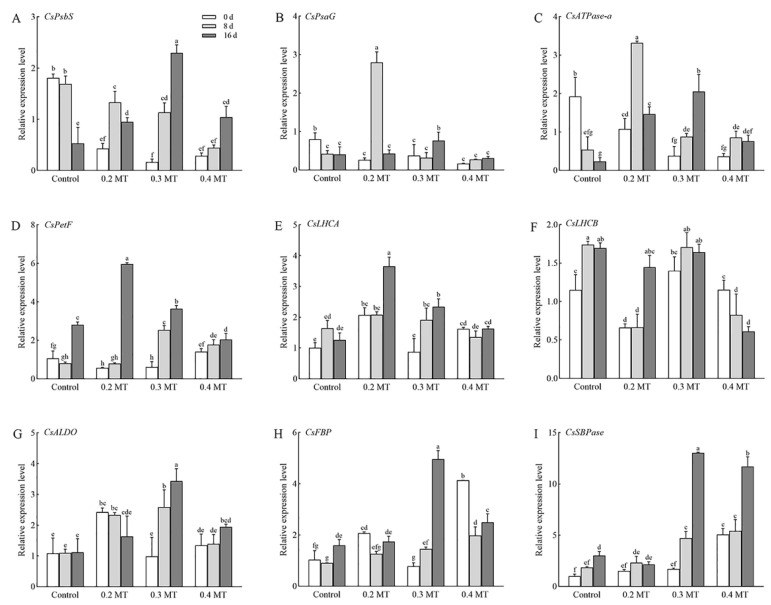
Expression validation based on RT-qPCR genes related to photosynthesis. (**A**) *CsPsbS*; (**B**) *CsPsaG*; (**C**) *CsATPase-a*; (**D**) *CsPetF*; (**E**) *CsLHCA*; (**F**) *CsLHCB*; (**G**) *CsALDO*; (**H**) *CsFBP*; (**I**) *CsSBPase*. Data are shown as means ± SD in three independent replicates. Different superscript letters indicate significant differences between different treatments at *p* < 0.05 level.

**Figure 9:**
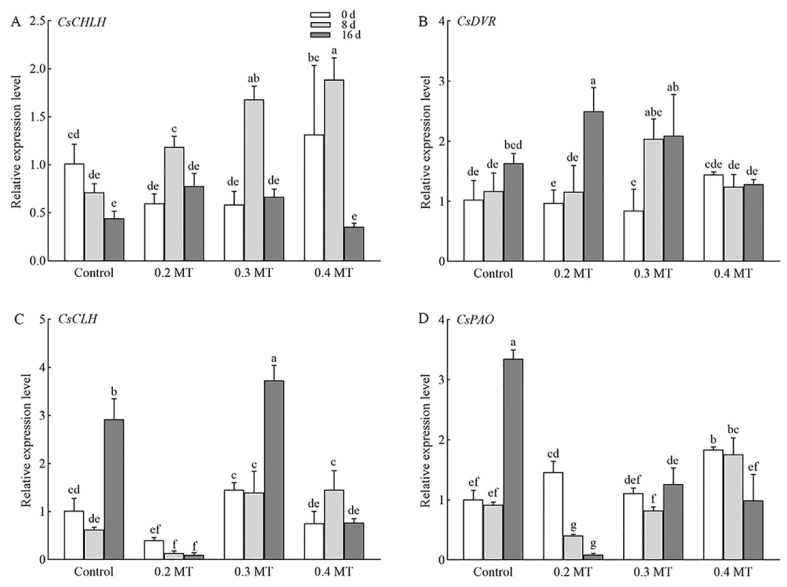
Expression validation based on RT-qPCR of genes involved in Chl metabolism. (**A**) *CsCHLH*; (**B**) *CsDVR*; (**C**) *CsCLH*; (**D**) *CsPAO*. Data are shown as means ± SD in three independent replicates. Different superscript letters indicate significant differences between different treatments at *p* < 0.05 level.

To elucidate the molecular mechanism of MT in photosynthesis of tea plants, we selected the genes involved in photosynthesis and Chl metabolism, and their expression profiles were determined (Figure 8 and Figure 9). Their nucleotide sequences are listed in Supplementary Table S1. Transcription of *CsPsbS* (*CSS0043679*) showed a pattern similar to those of *CsPsaG* (*CSS0047948*) and *CsATPase-a* (*CSS0018496*) under the 0.2-mM MT treatment, which had a high expression level at day 8, and then declined at day 16 (Figure 8A–C). Additionally, MT treatment at 0.2 mM significantly increased *CsPetF* (*CSS0040087*), *CsLHCA* (*CSS0038112*), and *CsLHCB* (*CSS0010537*) expression at day 16 (Figure 8D–F). *CsALDO* (*CSS0028383*) and *CsSBPase* (*CSS0032234*) remained at a relatively stable expression (Figure 8G,I). Compared to the control, the expression of *CsLHCB* (*CSS0010537*) and *CsFBP* (*CSS0042699*) were inhibited at day 8 under the 0.2-mM MT treatment (Figure 8F,H). The genes *CsPsaG (CSS0047948*), *CsPsbS* (*CSS0043679*), *CsATPase-a* (*CSS0018496*), *CsPetF* (*CSS0040087*), *CsALDO* (*CSS0028383*), *CsFBP* (*CSS0042699*), and *CsSBPase* (*CSS0032234*) exposed to 0.3 mM MT were significantly upregulated over time (Figure 8A–D,G–I). The upregulation of *CsLHCB* (*CSS0010537*) of the control was inhibited by the 0.3-mM MT treatment at days 8 and 16 (Figure 8F). The expression patterns of *CsPsbS* (*CSS0043679*) under the 0.4-mM MT treatment were similar to that of *CsSBPase* (*CSS0032234*), in which the expression levels were significantly increased at day 16 in comparison with day 0 (Figure 8A,I). Treatment at 0.4 mM inhibited the downregulation of *CsPsaG* (*CSS0047948*) and *CsATPase-a* (*CSS0018496*) in comparison with control (Figure 8B,C). The transcript levels of *CsLHCB* (*CSS0010537*) and *CsFBP* (*CSS0042699*) were significantly downregulated under the 0.4-mM MT treatment (Figure 8F,H). 

The gene *CsCHLH* (*CSS0016317*) showed a similar pattern under different MT concentration treatments, which was increased at day 8 and decreased at day 16 (Figure 9A). *CsDVR* (*CSS0008645*) showed a relatively stable expression in control and 0.4-mM MT treatment groups, whereas its expression obviously increased under the 0.2- and 0.3-mM MT treatments at day 16 (Figure 9B). *CsCLH* (*CSS0004684*) and *CsPAO* (*CSS0042634*) are involved in the process of Chl degradation, and were upregulated in control group at day 16 (Figure 9C,D). MT treatment at 0.2 mM inhibited the increase in *CsCLH* (*CSS0004684*) expression, and its expression was relatively stable. The transcription of *CsCLH* (*CSS0004684*) was increased under the 0.3-mM MT treatment at day 16 and 0.4-mM MT treatment at day 8, respectively (Figure 9C). *CsPAO* (*CSS0042634*) had a lower mRNA abundance under the 0.2-mM MT treatment at days 8 and 16 compared to day 0 (Figure 9D). It remained relatively stable expression under the 0.3-mM MT treatment, but 0.4 mM MT significantly downregulated the expression at day 16 (Figure 9D).

## 3. Discussion

Chl is one of the main pigments in leaf tissues and it is important for capturing light energy in photosynthetic organisms. A change in environmental factors will lead to a change in chlorophyll content, and then a change in photosynthetic performance [22]. Variation in Chl concentration directly affects the photosynthetic rate. Melatonin may help maintain high photosynthesizing efficiency by maintaining the integrity of chlorophyll molecules [20]. Melatonin can downregulate chlorophyll degrading enzymes in *Arabidopsis* and *Begonia* [23]. In our study, the Chl content of tea leaves in the control group decreased gradually over time, consistent with the change in the nett photosynthetic rate, which might be caused by the adaptation of tea plants to environmental changes. In addition, with the extension of time, tea plants also grew and developed. This self-physiological regulation may have led to changes in parameters in the control group. Exogenous application of MT increased Chl content (0.2 and 0.3 mM) or delayed Chl degradation (0.4 mM). This indicates that melatonin treatment is associated with enhancing chlorophyll biosynthesis and slowing chlorophyll decomposition. The reason may be that melatonin can maintain the stability of the chlorophyll structure and delay the degradation process of chlorophyll by affecting the activity of the key enzymes of chlorophyll biosynthesis and degradation. While a higher concentration (0.4 mM) delayed the degradation of chlorophyll, it may also be the self-stress caused by the high concentration of MT. This result was consistent with the findings in apple, rice, and kiwifruit [24,25,26]. The RNA-seq data also showed that 15 and 14 DEGs involved in Chl biosynthesis and cycle pathways were upregulated under the 0.2-mM MT treatment, respectively. These genes were considered as the candidate genes that regulated Chl biosynthesis in tea leaves. In addition, the expression of genes (downregulation of three *CLH* genes) related to the Chl degradation pathway caused Chl breakdown [27]. The pathway of Chl catabolism ‘PAO/phyllobilin’ occurs in all photosynthetic organisms [28]. MT treatment decreased or inhibited the transcription of *CsCLH* and *CsPAO*. 

The change in stomatal area and density regulate photosynthetic rate and transpiration [29]. The conductance and development of the stomatal were regulated by environmental changes, such as light, CO_2_ concentration, and plant hormone [30]. In the study, the decrease in *P*_n_ and *G*_s_ of the control was accompanied by a decline in *C*_i_, which indicated that the decrease in *P*_n_ was mainly due to stomatal limitation [31]. The application of 0.2 mM MT improved *P*_n_, *G*_s_, and *C*_i_. Previous studies have reported the photosynthetic rate in the tea leaves is small compared to many other tropical plants, being in the range of 2~14 μmol m^−2^ s^−1^ [32]. Further, the photosynthetic rate of tea leaves is closely related to tea varieties and leaf age, which is determined by its inherent genetic characteristics. The photosynthetic rate depends strongly on environmental conditions, such as irradiance, temperature, and nutrient supply, so that the photosynthetic rate changes with time and season [33]. The photosynthetic rate of tea leaves is relatively low in autumn. Additionally, the increase in stomatal area at day 16 and decrease in stomatal density from day 8 indicated that MT improved the photosynthetic capacity of leaves through stomatal regulation. With an increase in stomatal area, the inhibition of the gas and water exchange process was weakened [34]. This regulation mechanism helped reach a proper balance between water transport and photosynthesis, which maintained a photosynthetic capacity without losing excessive moisture. The lower the stomata density, the less moisture is lost through the leaves [34]. Stomatal density was negatively correlated with stomatal area, suggesting that plants adopted an ecological equilibrium strategy at the stomatal level to adapt to environmental cues [34]. We therefore hypothesized that MT could enhance photosynthesis in tea plants by short-term regulation of *G*_s_ and long-term regulation of stomatal development. 

Photosynthesis requires some major protein complexes, such as PSI, PSII, ATP synthase, etc. [35]. A total of 64 DEGs associated with these complexes were identified in our work, and 62 and 54 genes were upregulated at days 8 and 16 under the 0.2-mM MT treatment, respectively. These include genes involved in PSI(*PsaG*) and PSII (*PsbP*, *PsbQ*, *PsbR*, *PsbS*, *PsbY*, *Psb27*, and *Psb28*). PsbS proteins dissipate excess light energy by regulating non-photochemical quenching, a regulatory mechanism of reducing photoinhibition damage in plants, thereby protecting the PSII apparatus [36]. Based on the RT-qPCR, the expression level of *CsPsbS* decreased in the control group during treatment times, which might lead to the photoinhibition of PSII. Exogenous MT treatment at 0.2 and 0.3 mM partly protected and improved the activities of PSII by upregulating the expression of *CsPsbS*. The improvement in the photosynthetic electron transport process can provide NADPH and ATP for the dark reaction. PetF is an enzyme involved in the process of photosynthetic electron transport. Both the 0.2- and 0.3-mM MT treatment increased the transcription level of *CsPetF*. Plants develop LHC to reduce light absorption and protect PSII from excessive excitation [35]. LHC can absorb photons and transfers the energy to the center of the photosynthetic reaction to maximize the photosynthetic efficiency [37]. Compared with the control, six DEGs genes (*LHCA2,* two *LHCA4, LHCB1, LHCB3, LHCB6*) were upregulated. RT-qPCR showed MT treatment at 0.2 mM increased the expression of *CsLHCA* and *CsLHCB*, indicating that MT participated in the regulation of light energy capture. The improvement in photosynthesis-related gene expression in tea plants might be an important regulatory pathway for exogenous MT to enhance photosynthesis in tea plants.

The fluorescence level in plants is a probe of photosynthesis, which hides much important photosynthetic information [38]. *q*_P_ is the fraction of open reaction centers in PSII [39]. MT application increased the *q*_P_ level or alleviated the decline, which played an important role in enhancing electron transfer activity of PSII. *F*v/*F*m can be used to characterize the conversion efficiency of light energy in PSII reaction center, which is usually between 0.75 and 0.85. A decrease in *F*v/*F*m indicates PSII damage or photoinhibition [38]. It was found that ROS degrades the D1 protein of PSII center, destroys the structure of PSII, and leads to a decrease in *F*v/*F*m. ROS will also peroxidation with unsaturated acids (especially phospholipids) on the cell membrane to produce a large amount of MDA. MDA will further aggravate the oxidation reaction of cell biofilm and cause the destruction of biofilm structure [40,41]. A major function of melatonin may be to directly clear H_2_O_2_ and maintain it at stable levels [17]. Our results revealed that the *F*v/*F*m of the control was obviously reduced at day 16. The *F*v/*F*m level was still within the normal range, but the decrease in SOD activity and increase in MDA content manifested that the tea plants might be suffering oxidative damage at that time. The application of 0.2 mM MT significantly improved this situation. It was found that melatonin plays a role in scavenging ROS in various stress responses. Previous studies have confirmed that melatonin can enhance the activity of antioxidant enzymes and the accumulation of non-enzymatic antioxidants [42]. Similarly, in our study, 0.2 mM MT exogenous melatonin activated SOD activity and reduced the contents of H_2_O_2_ and MDA. The reason for this phenomenon may be that melatonin can improve the expression of plant antioxidant enzyme synthesis genes and enhance the ability of antioxidant protection system. SOD converts O^2−^ into H_2_O_2_, and POD, CAT converts H_2_O_2_ into harmless H_2_O and O_2_ [43]. When the effect of high concentrations of MT is reversed, it may be related to the stress caused by high MT concentration. The contents of H_2_O_2_ and MDA in tea plants treated by MT were decreased. These results suggested that melatonin helps to reduce membrane damage caused by ROS accumulation. The reason may be that exogenous melatonin has essential groups for scavenging reactive oxygen species, and melatonin can react with free radicals of reactive oxygen species to remove ROS together with protective enzymes. Melatonin itself is an efficient oxidant. It was found that after being absorbed by tea plants, melatonin reacted directly with ROS molecules such as H_2_O_2_ in cells, so as to eliminate ROS molecules and reduce MDA production. Antioxidant enzymes are distributed in various parts of plant cells and work together to remove ROS. SOD, POD, CAT, APX are the most important antioxidant enzymes in tea plant. Through a division of labor, they can effectively decompose excess O^2-^ into H_2_O_2_ and then into H2O [44]. Aghdam et al. found that melatonin can increase the activity of antioxidant enzymes in the leaves of Anthurium under low temperature storage, so as to reduce the contents of H_2_O_2_ and MDA [45]. The positive effect of exogenous MT on *F*v/*F*m, ROS scavenging and SOD activity suggested that MT might alleviate PSII inhibition and partially display direct antioxidant effect [46]. 

The physiological and metabolic processes in higher plants are complex. The effects of melatonin on the growth and development of tea plants need further study. With the further development of integrative omics analysis and deep genome sequencing of tea plants and other higher plants [4,47,48,49,50], new ideas and schemes will be provided for the effects of melatonin on photosynthetic characteristics and growth and development of tea plant and other higher plant.

## 4. Materials and Methods

### 4.1. Plant Materials and Exogenous Melatonin Treatments

The experiment was conducted in September 2020. Two-year-old vegetatively propagated tea plants (*Camellia sinensis* L. cv. ‘Zhongcha 108’) were used as experimental material. They were grown at the Tea Science Research Institute of the Nanjing Agricultural University (Nanjing, China). The cultivation substrate of the tea plants was composed of organic soil, vermiculite, and perlite (3:2:1, *v*/*v*). The tea plants were divided into four groups and sprayed with various concentrations of MT: 0 (control), 0.2, 0.3, and 0.4 mM every day, 16 times in total, 200 mL each time, and sprayed evenly on the front and back sides of the tea leaves with the sprayer. The standard was that the liquid was attached to the leaf surface but did not drip. The control group was sprayed with the same amount of distilled water. One hundred and fifty healthy tea plants were selected for each treatment. The first leaf and the second leaf of each treatment tea plants were collected at days 0, 8 and 16, respectively. Samples of each treatment were selected from 12 tea plants, mixed, and divided into three biological replicates. The harvested samples were used for physiological indicator measurement or stored at −80 ℃ for gene expression or transcriptome analysis.

### 4.2. Determination of Chl a, b and Total Chl Contents

Chl was extracted from the leaf samples with acetone, anhydrous ethanol, and distilled water, the volume ratio was 4.5:4.5:1.0. After removing the leaf veins, the leaves were cut into pieces, weighed at 0.1 g of tea leaves, 10 mL of extraction solution was added, and extracted in the dark for 24 h until the leaves were completely white. The mixed extract was used as a blank control, the absorbance values of each sample were measured by a microplate analyzer at 645 and 663 nm, respectively. The concentrations of Chl a, b, and total Chl were calculated according to Yang et al. [51]. Three independent biological replicates were performed for the determination of Chl a, b, and total Chl contents.

### 4.3. Determination of P_n_, G_s_, C_i_, and T_r_

For each treatment, nine tea plants were randomly selected and labeled, and the second leaf from the top was measured for each tea plant. The photosynthetic parameters (*P*_n_, *G*_s_, *C*_i_, *T*_r_) were determined by photosynthesis system (Li-6400, LI-COR, Inc. Lincoln, NE, USA) at days 0, 8 and 16, respectively. The determination conditions were as follows: light intensity 600 μmol·m^−2^·s^−1^, CO_2_ concentration 400 μmol·mol^−1^, leaf chamber temperature (25 ± 1) °C, relative humidity 70%~80%. Each measurement time was 9:00. The determination was repeated 3 times for each tea plant leaf.

### 4.4. Stoma Observation

The lower epidermis of tea leaves were stripped to observe stomatal [52] and measured by the nail polish blotting method, and the slides were observed under BX-53 fluorescence microscope (Olympus, Tokyo, Japan). Three tea plants were randomly selected from each treatment, and the second leaf was measured from each tea plant. CellSens Standard software was used to take photos and measure stomatal diameter. The calculation formula of stomata density and area referred to in Zhu et al. [34].

### 4.5. Measurement of Chl Fluorescence Parameters

The Chl fluorescence parameters of the tea leaf were determined by multifunctional modulation fluorescence imaging system IMAGING-PAM (IMAG-MAXI, Effeltrich, Germany). Before the measurement, the tea plants were exposed to darkness for at least 30 min, and the whole process was carried out in the dark [53]. The minimal Chl fluorescence (*F*o), maximal fluorescence (*F*m), and maximum photochemical quantum yield of PS II (*F*v/*F*m) was measured by IMAGING-PAM under dark adaptation. Subsequently, the light intensity of the photochemical stage was set as 600 μmol·m^−2^·s^−1^, and when the fluorescence was constant, intensive flash (saturated pulse light, 4000 μmol·m^−2^·s^−1^, 0.8 s) was applied. Other fluorescence parameters were obtained, such as minimal Chl fluorescence (*F*o’), maximal fluorescence (*F*m’), steady state fluorescence (*F*), photochemical quenching coefficient (*q*_P_) = (*F*m’ − *F*)/(*F*m’ − *F*o’), non-photochemical quenching coefficient (*q*_N_) = (*F*m − *F*m’)/(*F*m − *F*o’), and non-photochemical quenching (NPQ) = (*F*m − *F*m’)/*F*m’.

### 4.6. Determination of H_2_O_2_ and MDA Contents

Potassium iodide (KI) was used to quantify H_2_O_2_ content in accordance with the method described by Alexieva et al. [54]. Briefly, tea leaves of 0.1 g were ground in 1 mL 0.1% trichloroacetic acid (TCA) and centrifuged to obtain the supernatant for subsequent experiment. The reaction solution included 1 M KI solution, 0.1 M potassium phosphate buffer (pH 7.8) and supernatant. The control was 0.1% TCA. After reacting in darkness for 1 h, the absorbance at 390 nm was determined by a quartz plate. The H_2_O_2_ content in the sample was calculated according to the standard curve of known concentration of H_2_O_2_. Based on the principle and method mentioned by Hodges [55] and Tsikas [56], the assessment of lipid peroxidation was determined by MDA measurements. MDA is regarded as a component of thiobarbituric acid-reactive substances (TBARS). Tea leaves of 0.2 g were ground in 1 mL 5% trichloroacetic acid (TCA), and the homogenate after grinding was centrifuged at 12,000 rpm for 20 min to obtain the supernatant. Then, 1 mL supernatant and 1 mL 0.67% thiobarbituric acid (TBA) were mixed, and pre-warmed at 100 °C in a water bath for 30 min. The absorbance of supernatant was recorded at 450, 532, and 600 nm, respectively. All measurements were repeated independently three times.

### 4.7. Determination of Antioxidant Enzyme (SOD) Activity

Referred to the methods described by Wang et al. [24] for enzyme activity determination with slight modifications. In brief, tea leaves of 0.2 g were ground in 1 mL phosphate buffer (PBS, pH 7.8). The obtained homogenate was centrifuged, and the supernatant was the enzyme solution. The photochemical reduction of nitro blue tetrazolium (NBT) was used for the measurement of SOD activity. The total reaction system included 3 mL: 1.5 mL 50 mM phosphate buffered saline (PBS, pH 7.8), 300 μL 14.5 mM methionine, 2.25 mM NBT, 30 μM ethylenediaminetetraacetic acid disodium salt, 60 μM riboflavin each, 250 μL distilled water, 50 μL enzyme solution. PBS was used instead of enzyme solution as light control. The reaction started under the light, and it was allowed to run until the color turned purple. The absorbance of the reaction liquid at 560 nm was determined by a microplate reader. All measurements were repeated independently three times.

### 4.8. Transcriptome-Based Identification of Chl Biosynthesis and Photosynthesis-Related Differentially Expressed Genes (DEGs)

Our previous work had established transcriptome data at days 8 and 16 under the 0.2-mM MT treatment [57]. Herein, we screened the DEGs involved in Chl metabolism and photosynthesis. The Fragments Per Kilobase of exon model per million mapped fragments (FPKM) method was used to transform the gene expression in each sample, and the heat map was drawn to visualize the expression level by TBtools software (Guangzhou, China) [58].

### 4.9. RNA Isolation and Gene Expression Analysis

Total RNA was extracted from tea plant samples. First-strand cDNA was synthesized from the isolated total RNA with a Prime Script RT reagent kit (TaKaRa, Beijing, China) as instructed by the manufacturer. The quantitative real-time reverse transcription–polymerase chain reaction (RT-qPCR) was performed with Hieff qPCR SYBR Green Master Mix (Yeasen, Shanghai, China) on CFX96 Real-Time System (Bio-Rad, Hercules, CA, USA). Specific primers (Table 3) used for RT-qPCR assays were designed by Primer Premier 5.0 software (Vancouver, BC, Canada). *CsACT7* acted as an internal control gene [57,59]. The reaction mixture contained 1 μL of each primer, 10 μL of enzyme, 1 μL of diluted cDNA and 7 μL of double-distilled water. The reaction conditions were set as follows: 95 °C for 30 s, followed by 40 cycles of denaturation at 95 °C for 5 s, and 55 °C for 25 s. Relative gene expression was calculated following the 2^−∆∆CT^ method based on Pfaffl [60] mentioning. Total RNA isolated in tea leaves were repeated independent at least three times.

### 4.10. Statistical Analysis

All statistical analysis was conducted using SPSS 25 software (Chicago, IL, USA). Each value was expressed as the mean ± standard deviation (SD). Variance (ANOVA) was used to perform data analysis, and significant differences were detected by Duncan’s multiple range tests at a probability level of 0.05. GraphPad Prism 8.0 software (San Diego, CA, USA) was used to plot charts.

## 5. Conclusions

**Figure 10:**
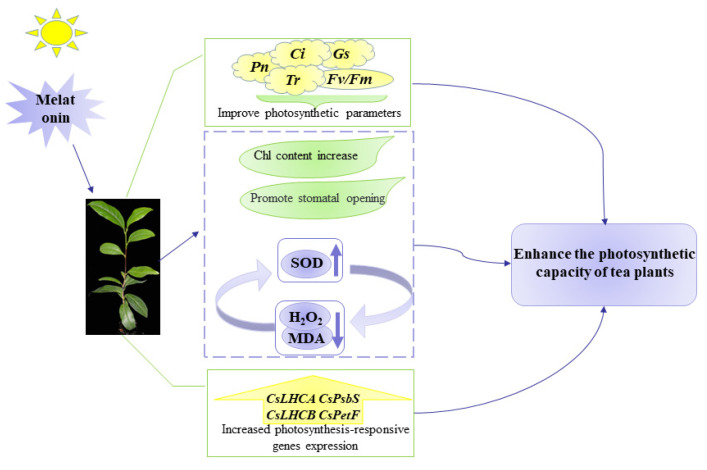
Possible mode of exogenous melatonin enhancing photosynthetic capacity of tea plants.

A possible mode network model based on the current study is shown in Figure 10. The application of 0.2 mM MT increased Chl contents and improved *P*_n_, *G*_s_, *C*_i_, and *T*_r_ in tea leaves. Under MT treatment, the chlorophyll fluorescence level and SOD activity of tea plants were increased, and the contents of H_2_O_2_ and MDA were inhibited, indicating that exogenous MT participated in the regulation of photochemical efficiency of PSII. Transcriptome data based on RNA-seq and gene validation measured by RT-qPCR showed that MT could regulate the gene expression involved in photosynthesis and Chl metabolism. In general, our findings provide a reference for further exploring the role of MT in photosynthetic capacity of tea plants.

## Figures and Tables

**Table 1:** Effect of exogenous melatonin on chlorophyll *a, b*, and total chlorophyll contents in tea leaves.

Treatments	Chl a Content (mg/g FW)			Chl b Content (mg/g FW)			Total Chl Content (mg/g FW)		
	Day 0	Day 8	Day 16	Day 0	Day 8	Day 16	Day 0	Day 8	Day 16
Control	1.073 ± 0.008 ^b^	0.967 ± 0.020 ^d^	0.975 ± 0.042 ^d^	0.593 ± 0.004 ^c^	0.497 ± 0.004 ^f^	0.427 ± 0.005 ^g^	1.667 ± 0.001 ^b^	1.464 ± 0.018 ^f^	1.403 ± 0.046 ^g^
0.2 MT	0.980 ± 0.031 ^d^	1.072 ± 0.001 ^b^	1.115 ± 0.013 ^a^	0.388 ± 0.006 ^g^	0.550 ± 0.036 ^de^	0.718 ± 0.012 ^a^	1.368 ± 0.026 ^gh^	1.621 ± 0.035 ^c^	1.832 ± 0.025 ^a^
0.3 MT	1.037 ± 0.002 ^c^	0.867 ± 0.007 ^e^	1.089 ± 0.010 ^ab^	0.477 ± 0.009 ^f^	0.473 ± 0.013 ^f^	0.718 ± 0.012 ^a^	1.515 ± 0.007 ^e^	1.340 ± 0.006 ^h^	1.807 ± 0.002 ^a^
0.4 MT	1.099 ± 0.006 ^ab^	1.017 ± 0.005 ^c^	0.896 ± 0.009 ^e^	0.532 ± 0.011 ^e^	0.562 ± 0.007 ^d^	0.680 ± 0.016 ^b^	1.631 ± 0.004 ^bc^	1.579 ± 0.001 ^d^	1.576 ± 0.011 ^d^

Data are shown as means ± standard deviation (SD) in three independent replicates. Different superscript letters indicate significant differences between different treatments at *p* < 0.05 level.

**Table 2:** Effect of exogenous melatonin on *F*o, *F*m, and *F*v/*F*m in tea leaves.

Treatments	*F*o			*F*m			*F*v/*F*m		
	Day 0	Day 8	Day 16	Day 0	Day 8	Day 16	Day 0	Day 8	Day 16
Control	0.109 ± 0.005 ^e^	0.141 ± 0.011 ^a^	0.142 ± 0.005 ^a^	0.516 ± 0.020 ^e^	0.688 ± 0.043 ^a^	0.629 ± 0.034 ^b^	0.789 ± 0.002 ^ab^	0.795 ± 0.003 ^a^	0.775 ± 0.005 ^cde^
0.2 MT	0.133 ± 0.004 ^bc^	0.129 ± 0.009 ^bcd^	0.125 ± 0.005 ^d^	0.580 ± 0.014 ^cd^	0.595 ± 0.038 ^cd^	0.602 ± 0.019 ^c^	0.771 ± 0.002 ^e^	0.783 ± 0.002 ^bcd^	0.793 ± 0.001 ^a^
0.3 MT	0.127 ± 0.007 ^cd^	0.133 ± 0.013 ^bc^	0.141 ± 0.007 ^a^	0.567 ± 0.025 ^d^	0.588 ± 0.032 ^cd^	0.591 ± 0.023 ^cd^	0.776 ± 0.004 ^de^	0.775 ± 0.012 ^de^	0.761 ± 0.006 ^f^
0.4 MT	0.111 ± 0.005 ^e^	0.135 ± 0.009 ^ab^	0.135 ± 0.008 ^ab^	0.517 ± 0.023 ^e^	0.521 ± 0.052 ^e^	0.527 ± 0.032 ^e^	0.786 ± 0.001 ^abc^	0.738 ± 0.032 ^g^	0.743 ± 0.008 ^g^

Data are shown as means ± standard deviation (SD) in three independent replicates. Different superscript letters indicate significant differences between different treatments at *p* < 0.05 level.

**Table 3:** Primer sequences of photosynthesis and Chl metabolism related genes for RT-qPCR.

Transcriptome ID	Gene Symbol	Molecular Function	Forward Primer (5′-3′)	Reverse Primer (5′-3′)
CSS0047948	*CsPsaG*	Photosystem I subunit G	GTCATACCGTTCTGTTCG	GCACTGGTCTGTCCCT
CSS0043679	*CsPsbS*	Photosystem II subunit S	TCTCACATCTTACTACCTCCAC	AAACCCAATCCCACCC
CSS0018496	*CsATPase-a*	F-type H^+^-transporting ATPase subunit a	ATCAATACTCGCTTCGC	CATCCTGGTCTTCCTCT
CSS0040087	*CsPetF*	Ferredoxin	TGGGATGATGGTTTCTG	TGAGCACGTACCCTTTC
CSS0038112	*CsLHCA*	Light-harvesting complex I chlorophyll a/b binding protein	GGACCCTGAGAACCTAAA	GACGATGACGCGAAGTA
CSS0010537	*CsLHCB*	Light-harvesting complex II chlorophyll a/b binding protein	CGGAAAGGCGGTGAAA	CAGACAATGGACCCAAATAC
CSS0028383	*CsALDO*	Fructose-bisphosphate aldolase	AAAGCGAGGAAGAGGC	CCGAATGAGAACGAAAGT
CSS0042699	*CsFBP*	Fructose-bisphosphatase	GTATTTCAATGGCGTGTA	TTATCCCTGTCCTCCC
CSS0032234	*CsSBPase*	Sedoheptulose-bisphosphatase	TGAGGCTGCTGTTTGA	ATCGGATAATCTCGTTCT
CSS0016317	*CsCHLH*	Magnesium chelatase H subunit	GCTGCTTACTACTCGTTT	CTCATTCCCACCTGCT
CSS0008645	*CsDVR*	Divinyl chlorophyllide a 8-vinyl-reductase	TGGGTGGACAGGTTGA	CACAGTCTGCGATAAATG
CSS0004684	*CsCLH*	Chlorophyllase	ATCATCACCACCTAAACC	CTGAGGAGCAACGACTA
CSS0042634	*CsPAO*	Pheophorbide a oxygenase	GACTTGACCCTGTTGC	AAGCCAGTACCGATGA

## Data Availability

In this section, transcriptional data, physiological and anatomic metabolic data were measured by the authors themselves.

## Supplementary Materials

The following supporting information can be downloaded at: https://www.mdpi.com/article/10.3390/ijms23126694/s1.
