# Supplementary material for: Exogenous Melatonin Enhances Photosynthetic Capacity and Related Gene Expression in A Dose-Dependent Manner in the Tea Plant (Camellia sinensis (L.) Kuntze)
Source: Int J Mol Sci. 2022 Jun 15;23(12):6694. doi: 10.3390/ijms23126694 (PMC9223723; doi:10.3390/ijms23126694)
Supplement: Supplementary file 1 [file ijms-23-06694-s001.zip › ijms-1745059-supplementary.pdf]

SupplementaryTable S1 Nucleotide sequences of the selected DEGs for gene expression analysis

Gene symbol	Transcriptome ID	Nucleotide sequence
CsPsaG	CSS0047948	ATGAAGAACAAACAAGAAAATTGTAGCCTCCCCACCTATGCACATGAATTACTAGCACAACCCAACAACAATCCAAATTCAAATTCAAATTC<br>AAATTCAAATTCAAATTCATCCAAAGAACTTGGGATCATATCCATTGCTAGAGGTAGCAAGTATATAGTAAGCAACGATGTGTCCAATGGAAC<br>CCCAAGCCAAGACATCGACGATGTTGAACCCAACTGGGTCGTTGGATTTGAGGAGACTCACATACTCTTTGGCTCGAGAATCACCAGCTTC<br>GAAATGGGTCATACCGTTCTGTTCGGGAACCTGTTTGGCCACATTCTCTCTCTGGAAATTGAAGAACACGAACCTGCCCAAAAACAGGGAC<br>AGACCAGTGCTGAGACTGATGACCAGAGATGTGTTCAATTCGGCTTTAATGGCGCCGCTGCATCGTTTTTTGGGGGTGAATTTGACAGTGGA<br>TATGGATTTGGTGAGGGAGAGTGGGGATTTGGTGGTGGGTCTGAGGCCTTGGAAGGTGGGTGGTGA
CsPsbS	CSS0043679	ATGGCTCAGACCATGTTGCTCACTTCTAGTTCAAGTGTCTATAGTCATGCATTGGATTTGAAGAGACAACCATTACTTGAAAGTCTAAGGCCC<br>AAACCATTCTCTCACATCTTACTACCTCCACTTCCATCTTCTTCTTCATTTCATCAGACTACCACCATTGCTCTCTTCAAATCCAAAACCAAAG<br>CTGCCCCTGTCAAGAAGGTTGCCGAGCCTAAGCCGAAGGTTGAAAGTGGTATTTTTGGCACGTCGGGTGGGATTGGGTTTACAAAGCAAAA<br>TGAGCTTTTTGTGGGTCGTGTTGCCATGATCGGCTTCGCTGCATCTTTGTTGGGAGAAGCAATAACAGGGAAAGGAATCCTAGCACAACTGA<br>ATCTGGAGACTGGAATTCCCATCTATGAAGCTGAGCCTCTTCTCCTCTTCTTCATCCTTTTCACCCTCCTCGGAGCCATCGGAGCTTTGGGCG<br>ATAGGGGTCGCTTTGTTGATGACCCCCCTACCGGGCTTGACAAGGCTGTCATCCCTCCAGGCAAAAGTCTCAGATCAGCATTGGGTCTTAAA<br>GAAGGAGGTCCACTATTTGGATTCACAAAGTCGAATGAGCTGTTCGTGGGACGATTGGCTCAGTTGGGAATTGCATTCTCTATAATTGGAGA<br>GATAATCACGGGGAAAGGAGCTCTGGCACAGCTAAACATCGAGACAGGAGTTCCAATCGGCGATATTGAGCCCCTTGTGTTGTTCAATGTCC<br>TCTTCTTCTTTGTTGCCGCATTGAATCCTGGAACTGGCAAGTTTCAAACAGATGAAGAAGAGTAG
CsATPase-a	CSS0018496	ATGAATGTTCTATCATGTTCCATCAACACACTAAAAGGTTTATATGATATATCCGGTGTGGAAGTAGGCCAACATTTCTATTGGAAAATAGGAA<br>GTTTCCAAGTCCACGGCCAAGTACTTATTACTTCTTGGGCTGTAATTGCTATTTTATTAGGTTCAGCCACTATAGCTGTTCGGAACCCACAAAC<br>TGTTCCGACTGGCGGTCAGAATTTCTTCGAATATGTCCTTGAATTCATTCGAGATGTGAGTAAAACCCAAATTGGGGAAGAATATGGTCCTTG<br>GGTTCCCTTTATTGGAACTATGTTTCTATTTATTTTTGTTTCGAATTGGTCAGGAGCTCTTTTACCTTGGAAAATCATACAATTACCTCATGGGG<br>AGTTAGCCGCACCCACGAATGATATAAATACTACTGTTGCTTTGGCTTTACTCACGTCAGTGGCATATTTCTATGCGGGTCTTAGCAAAAAAG

		GATTGGCTTATTTTGGGAAATACATTCAACCAACTCCAATCCTTTTACCCATTAACATCTTAGAAGATTTCACAAAACCTTTATCACTTAGTTT<br>TCGACTTTTCGGGAATATATTAGCCGATGAATTAGTAGTTGTTGTTCTTGTTTCTTTAGTACCTTTAGTGGTTCCTATACCTGTCATGTTTCTTG<br>GATTATTTACAAGTGGTATTCAAGCTCTTATTTTTGCAACTTTAGCCGCGGCTTATATAGTAAACTCTCCTGGGGCCATAGCCGAGTTCCGAAA<br>GGATTACCAAATTCCAGACGATGTCCATCTTTTCCTGGCCAAACTAGATGCCACGCCCTGGGGAAAACAAGGTTTCGTTCCATTTACCGTACT<br>ATCCATTCTACTCCTTTGTGAATTCCTTAGGCAAACAAAACTCTGCCCCACTCAGCTCTCCACAAACTCATATAGAATAATAAATGGCATCGC<br>CGAGCTAAACCGCCGGCTTGGCCTCAACCTCGATTTAGCCGAGCTATTTCATCAATACTCGCTTCGCCAAAATGAAGACAGCTGGGAAGCCC<br>TAATACCGGGATACGGAATAAACTGGAATAAAGGATACCAATACCCCAATTTAGTCGCACTCCGCGCTGCACTTCGGCACCGCGATCGAGGT<br>TGCGCCGAGCTACTCAATTTTGAACCGGCATACAGATACAATAGTAAGAGGAAGACCAGGATGGCAAAGCGTCGAGCTGTGCGAGCCCTAA<br>TCACCGAGACAACTTCAATTCCAAATAGCGTGGTTCAAACTGAGACAAGTCCAGCTGGTCAAACAGTTGATGCAGTGATGGCCCTCCCAAC<br>AGCCCAAGCCTTGTCATCCCGGTCGAGGAAGCGCGCTCGCACTCCCTCAATCGAGCCACAACACCTTGTGGATGACGACGACACCTTACTC<br>CCTCAGTTTACGCGACCGAGCCCAGAGCCCGTGGGTTCTGACCGAGCTAGCCCTTCTGAATGGGCACCAAAAATAACTTTCAAAAACCGAG<br>CCGTAACAAACACAGACTCGGTGGTTACAGAAAAAGACCATTCTCTTGCCTTTAACCTCACCAAGAGCGTGTACCTTCCAGCCAATATGGA<br>GCGTCACGAACATCTGACCAAGCTGAAAGCCATCCGCTCGGCAACAAAGTCGATGGTTCTGGTAATTTCTTCTTTTGCTTATCTTCCCAATTC<br>GCACATCTGTTTGCACGGTTTTGATCATAACTGTTATCCCTCCTTTGTAGGCCTTGCAGAAGAATCACATTGCTCATAA
CsPetF	CSS0040087	ATGTCGACTGTGACAATTCCTTCACCTTGCTTGTTCAAAAGTGCACCTGTAACCAGATCCTCTGCTTTGATCAAGAATCCAAAGTCTTTGGGT<br>TCAGTAAAGAGTGTTTCCAAGTCATTTGGCATGAAGTCCCCCTCCTCCTTCAGAGTATCTGCCATGGCAGTGTACAAAGTGAAACTGATTGG<br>ACCCGATGGCGAAGAGAACGAGTTTGATGCCCCCGATGATACATACATCCTTGACTCAGCTGAAAACGCAGGACTGGAGTTGCCCTACTCG<br>TGCAGGGCTGGTGCCTGCTCTACGTGCGCTGGGATGATGGTTTCTGGATCCGTGGACCAATCAGACGGTTCATTTCTTGATGATAATCAGATG<br>AAGAAAGGGTACGTGCTCACCTGCGTTTCGTACCCCACTTCTGATTGTGTGATTCAAACTCATAAAGAAAGTGAACTTTATTGA
CsLHCA	CSS0038112	ATGGCAACCATTACGGCGCAAGCATCCACCGCTGTCTTCCGGCCACGTGCCGCCAAATCCCAGTTCCTTACCGGTTCTTCCGGCAAGCTCAA<br>CAGAGAAATTTCACTTAAATCAAAATCTTCATCACCAAGATCATTCAAAGTTGAAGCCAAAGGTGAATGGTTACCGGGATTGCCCTCACCAG<br>ACTACCTTAATGGCAGTCTCCCTGGTGACAATGGATTTGATCCTCTAGGCCTTGCAGAGGACCCTGAGAACCTAAAATGGTACATCCAAGCC<br>GAGCTTGTGAACAGCCGGTGGGCCATGTTAGGGGTCACCGGAATGCTGCTGCCAGAAGTGTTCTCAACTATCGGAATAATCAATGTCCCCA<br>AATGGTACGATGCAGGAAAAGCCGAGTACTTCGCGTCATCGTCAACTCTGTTCATCCGACGATGGCAGGACATCAAGAACCCAGGAAGTGT

		CAACCAAGATCCTATCTTCAAGAACTATAGCTTGCCTCCCGGTGAGGTTGGGTACCCTGGTGGCATTTTCAATCCCCTGAATTTTGCACCCAC<br>CCAGGAGGCCAAGGAGAAGGAGCTTGCCAACGGGAGATTGGCAATGTTGGCATTCTTGGGATTCATCGTTCAGCACAATGTGACCGGAAA<br>AGGGCCATTCGACAACCTCTTGCAGCATCTCTCCGACCCATGGCACAACACAATTATTCAGACATTCCAGGGTTACTAA
CsLHCB	CSS0010537	ATGGCTGCTTCTACCATGGCTCTCTCTTCCCCATCTTTCGCCGGAAAGGCGGTGAAACTTGCCCCGGAGGTTCTTGGTGGTGGACGGATCAG<br>TATGAGGAAGACCGGCAAGCAAGTCCCATCTGGAAGCCCTTGGTACGGTCCAGACCGAGTCTTGTATTTGGGTCCATTGTCTGGTGATCCCC<br>CATCTTACCTCACTGGAGAATTCCCTGGTGATTATGGTTGGGACACTGCTGGGCTTTCAGCCGATCCAGAAACTTTTGCCAAGAACCGTGAG<br>CTTGAGGTGATTCACTGCAGATGGGCCATGCTTGGAGCTTTGGGTTGCGTCTTCCCCGAGCTTTTGGCCCGCAACGGTGTCAAGTTCGGCGA<br>GGCTGTGTGGTTCAAGGCCGGTGCCCAAATCTTCAGCGAGGGTGGGCTTGACTACTTGGGCAACCCTAGCTTGATCCATGCCCAAAGCATT<br>CTGGCCATCTGGGCTTGCCAAGTTATCTTGATGGGGGCTGTTGAGGGTTACCGTATTGCCGGTGGGCCACTAGGAGAGGTGACAGACCCGCT<br>CTACCCCGGCGGAAGCTTTGACCCATTGGGCCTAGCTGATGATCCAGAGGCTTTTGCAGAGCTCAAGGTGAAGGAGATCAAGAACGGAAG<br>ACTGGCCATGTTCTCAATGTTTGGGTTCTTTGTTCAGGCCATTGTGACAGGAAAGGGACCATTGGAGAACCTGGCTGACCATCTTGCTGACC<br>CAGTTAACAACAATGCTTGGGCTTATGCTACCAACTTTGTCCCCGGCAAGTGA
CsALDO	CSS0028383	ATGTCGGCCTTCTTAGGAAATTATGCAGAGGAACTAATCAAGAATGCCAAGTACATTGCCACCCCCGGCAAGGGCATTTTGGCCGCGGATGA<br>AAGCACTGGCACCATTGGTAAACGTCTGTCGAGCATCAGCGTTGAGAACACCGAGCACAACCGCCAGTCCCTCCGCGAGCTCCTCTTCACC<br>GCTCCTAATGCCCTCCCCTACCTCTCTGGTGTCATTCTCTTCGAGGAGACCCTCTACCAAAAAACCTCTGACGGAAAGCCTTTCGTCGAGCT<br>CCTTAAGGAGAACAATGTCATTCCAGGTATCAAAGTCGACAAGGGTGTTGTTGAATTGGCCGGCACCAATGGCGAGACCACCACCCAGGGC<br>TTCGACTCGCTTGGATCCCGGTGTCAGCAGTACTACAAGGCCGGTGCACGCTTTGCCAAGTGGCGTGCCGTCCTCAAGATAGGCCCCACCG<br>AGCCATCCGAACTGTCAATCCAGCAGAATGCCCAGGGGCTGGCTCGCTATGCAAGGATATGTCAAGATTGGGGACTTGTCCCAATTGTTGAG<br>CCAGAGATTCTAACTGATGGAAACCATGACATCAAGAAATGTGCAGCTGTGACTGAAACTGTGCTTGCAGCTGTCTACAAGGCACTCAATG<br>ATCACCATGTTCTTCTTGAAGGAACACTTTTGAAACCCAACATGGTCACACCAGGCTCTGATAGCCCAAAGGTGACACCTGAGGTGATCGC<br>CGAACACACAGTTTTAGCATTGCGACGAACAGTTCCACCTGCAGTACCCGGGATTGTGTTTTTGTCAGGTGGGCAAAGCGAGGAAGAGGC<br>AACCCTCAACCTGAATGCCATGAACAAGTTAGACTTGTTGAAGCCATGGACACTTTCGTTCTCATTCGGGCGAGCTCTGCAGCAGAGCACA<br>CTCAAGACATGGGGTGGGAAGAAGGAGAATGTTGGGAAAGCTCAAGAGACATTCTTGCTAAGGTGCAAGTCCAATTCTGAGGCCACTTTG<br>GGGAAGTATGGTGGTGGTGGTGGTGGTGGTTTGGCTTCTGAGAGTTTGTTTGTTAAGGGTTACAAGTACTAA

CsFBP	CSS0042699	ATGGTTGCAACTCCAGCAGCAACATCCCCAGCCTCTCAGCTCCTCTTTTCGACCTCTCACTCCACCTCTCGTCTCTCCCCTTTCCAAGCATGC<br>GTCTTGGACGCCAAGACTCTCTTGTCGTGCCCCACGCGGCGCCAAGTGGTGGTGAGTGGTGGTGGAAGTGTGAGGTGCATGGCCGTGGGG<br>ACAGAATCGGAGACGGAGAAGAAGAAGAAGACTAGTAGTAGTAGTAAGTTTGAGATACAGACATTGACGGGTTGGCTGTTGAAGCAAGAA<br>CAGAAGGGGGTGATAGATGCTGAGCTCACGATAGTGATGTCGAGTATTTCAATGGCGTGTAAGCAGATTGCTTCGTTGGTTCAGAGAGCTAG<br>CATTTCCAACCTTACTGGTGTTCAGGGTGCTGTTAATATCCAAGGCGAGGACCAGAAGAAGCTCGATGTTGTCTCCAACGAGGTGTTCTCAA<br>CTTGTTTGAGGTCAAGTGGGAGGACAGGGATAATAGCATCAGAGGAAGAGGATGTACCAGTGGCAGTTGAAGAGAGTTACTCTGGTAACTA<br>CATTGTTGTTTTTGACCCTCTTGATGGATCTTCCAACATTGATGCTGCTGTCTCCACTGGCTCTATCTTTGGAATCTACAGCCCCAATGATGAG<br>TGTCTTGCTGATGTTGGTGATGATGATTCCACACTTGACAAAATAGAACAGAGGTGTATAGTAAATGTGTGCCAACCAGGAAGCAATCTTCT<br>TGCTGCTGGCTACTGCATGTACTCAAGCTCCATCATCTTTGTCCTCTCCATTGGAAATGGAGTTTTTGCATTCACTTTGGACCCCATGTATGGT<br>GAATTTGTGCTAACTCAAGAGAACATTCAGATACCGAAATCCGGGAAAATTTACTCATTCAATGAAGGAAACTACCAGTTATGGGATGACAA<br>GCTCAAGAAGTACATTGATGACCTTAAGGACCCCGGTCCTAGTGGCAAGCCTTATTCTGCTAGGTACATTGGCAGCTTGGTTGGTGACTTTC<br>ACCGGACTATGTTGTATGGTGGCATTTACGGGTACCCTAGCGACAAAAAGAGCAAGAATGGGAAGCTGAGGTTGTTGTATGAGTGTGCACC<br>GATGAGCTATTTGGCAGAGCAGGCGGGTGGCAAAGGGTCCGATGGTCATGTGAGGGTTCTTGATATCCAGCCAACTGAGATTCATCAGCGC<br>GTTCCACTTTACATTGGAAGCACAGAGGAAGTGGAAAAATTGGAGAAATATTTAGCCTAA
CsSBPase	CSS0032234	ATGGAGACTAGCATCACGTGCTGCGCACGTGGGTTCCTCCCTGCAGGTGTCTCTTCTCAACATTCAACTGCTTTTGTTTGTCAATCTTCCATT<br>TCTCCATCCTTCTGCTCCAAGAGTCTGAAATCGAGCTCACTATTTGGGGAGTCCTTGAGGATCATGTTAAAGGCATCATCAGTTAAGGTGTCA<br>AAGACCAACAACTCTTCCCTTGTAACCAGGTGCGAGATTGGTGACAGTCTGGAAGAGTTCCTTACAAAGGCATCTTCAGATAAGGGACTGA<br>TAAGATTAATGGTGTGCATGGGTGAAGCACTCAGAACCATTGCTTTCAAAGTGAGAACAGCTTCTTGTGGAGGAACAGCCTGTCTCAACTC<br>TTTTGGAGATGAGCAGCTCGCAGTTGACATGCTCGCCGATAAGCTCCTTTTTGAGGCCTTGACTTATTCCCACTACTGCAAATATGCTTGCTC<br>TGAGGAAGTCCCTGAACTCCAAGACATGGGAGGCCCAGCTCAAGGTGGATTCAGTGTTGCTTTTGACCCTCTTGATGGGTCTAGTATAGTGG<br>ACACAAATTTCACAGTTGGCACCATATTTGGGGTGTGGCCTGGAGACAAGTTAACAGGTGTGACAGGGAGAGATCAAGTTGCTGCAGCCAT<br>GGGGATTTACGGACCCCGAACTACATATGTTCTTGCTCTTAAAGACATGCCAGGCACTCATGAGTTCCTTCTTCTTGATGAAGGAAAATGGCT<br>ACAAGTCAAAGACACAACAGAAATTGGTGAAGGAAAGATGTTCTCTCCTGGAAATTTGAGAGCCACATCTGACAACCCTGACTATGACAA<br>GCTGATCAACTACTATGTAAGAGAGAAATATACATTGCGATACACTGGAGGAATGGTGCCGGATGTTAATCAGATAATAGTGAAAGAGAAAG

		GTATATTCACAAATGTGACATCCCCATCAGCGAAAGCAAAGCTGAGGCTGCTGTTTGAGGTGGCTCCTTTAGGGTTCTTAATTGAGAAAGCA<br>GGGGGATACAGTAGTGATGGCAAACAGTCTGTGCTTGACAAGGTGATTGTTAATCTTGATGACAGGACTCAAGTTGCTTATGGATCCAAGAA<br>CGAGATTATCCGATTTGAGGAAACACTATACGGTTCCTCCAGGCTCAAGACCGGTGTGCCAGTTAGCGCCGTTGCTGCTTAA
CsCHLH	CSS0016317	ATGGCTTCTTTAGTTTCTTCACCATTCACATTACCCACCTCAAAAACAGACCAACTATCTTCAATCTCTCAAAAACACTACTTTCTTCACTCTT<br>TCCTTCCCAAGAAAACCAACCAAACAAACCCAAAATCATCATCCATGAGAGTGAAATGTGCTGCAATTGGCAATGGACTATTCACCCAAAC<br>ATCCCCGGAAGTCCGCCGAATAGTCCCCGACAACATTCAAGGCCTCCCAACCGTGAAAGTGGTGTATGTAGTCCTTGAAGCTCAATACCAAT<br>CGTCCCTCTCCGCCGCGGTGCGTACTCTCAACAAAAATGGCAACTTTGCTTCATTTGAGGTTGTTGGGTACTTAGTTGAAGAGCTTAGAGAT<br>GAGAATACATACAAATCTTTCTGTAAAGACCTTGAGGATGCTAATGTATTCATAGGCTCATTGATTTTCGTTGAAGAGCTTGCTTTGAAGATCA<br>AGACTGCTGTTGAGAAAGAGAGGGACAGACTTGATGCAGTCTTGGTGTTCCCTTCAATGCCTGAAGTAATGAGACTCAACAAGTTGGGTTC<br>ATTTAGTATGTCCCAACTGGGGCAATCAAAGAGCCCCTTTTTTCAGCTATTCAAGAGAAAGAAACAGTCAGCTGGGTTTGCTGAGAGTATGC<br>TAAAGCTTGTGAGGACATTGCCCAAAGTACTAAAGTACTTGCCAAGTGATAAGGCTCAAGATGCCAGGCTATACATACTCAGTTTGCAGTTT<br>TGGCTTGGTGGGTCACCAGACAATTTGGTGAATTTCTTGAAAATGATTTCTGGGTCTTATGTACCGGCATTGAAAGGGATGAAAATCCAGTAT<br>TCGGACCCGGTTTTGTTCTTGGATAGTGGAATTTGGCACCCTTTGGCCCCATGTATGTATGATGATGTGAAGGAGTACTTGAATTGGTATGGG<br>ACTAGAAGGGATGCCAATGAGAGGATCAAGGGTCCAAATGCACCGGTGATTGGGTTGGTTTTGCAGAGAAGTCATATTGTTACCGGGGATG<br>AGAGTCACTATGTGGCTGTGATTATGGAATTGGAGGCAAAAGGGGCTAAAGTCATACCAATTTTTGCCGGTGGGCTTGATTTCTCGGGGCCA<br>GTTGAGAGGTTTTTCATCGATCCGATTACGAAGAAGCCGTTTGTGAATTCAGTGATCTCACTGACAGGTTTTGCTCTTGTTGGAGGGCCGGC<br>CAGACAAGATCATCCGAGGGCTGTTGAGGCACTGACTAAGCTTGATGTGCCTTACATTGTTGCAGTGCCTTTGGTGTTTCAGACAACAGAG<br>GAGTGGCTGAATAGCTCCTTGGGGTTACACCCAATTCAGGTGGCTTTGCAAGTTGCTCTTCCTGAGCTTGATGGAGGCATGGAGCCCATTGT<br>TTTTGCCGGGCGGGATCCTAGAACAGGGAAATCACATGCTCTTCACAAAAGGGTGGAGCAACTCTGCACCAGGGCAATCAGATGGGCTGA<br>ACTGAAAAGGAAGTCAAAGGCAGAGAAGAAGCTCGCAATTACTGTTTTCAGTTTCCCTCCAGACAAAGGAAACGTTGGAACTGCAGCATA<br>CCTGAATGTCTTCGCTTCCATTTACTCTGTGTTGAAGGACCTCCGAAAAGACGGTTACAATGTTGATGGCCTTCCAGAAACTTCCGAAGCCT<br>TAATTGAAGAAATCCTTCATGATAAAGAGGCTCAATTCAGCAGCCCAAATCTCAACGTAGCTTATAAAATGGGTGTCAGAGAGTACAAAAAT<br>TTGACTCCCTATGCCACATCATTGGAAGAGAACTGGGGGAAACCTCCTGGAAATTTGAATTCTGATGGGGAAAATCTGTTGGTCTACGGAAA<br>ACAGTATGGAAACGTTTTCATTGGTGTTCAGCCCACATTTGGTTACGAGGGTGATCCTATGCGGCTTCTTTTTGCCAAATCAGCTAGCCCACA<br>TCATGGGTTTGCTGCTTACTACTCGTTTGTCGAGAAAATTTTCAAAGCTGATGCTGTTCTTCATTTTGGCACTCACGGTTCGCTTGAATTCATG

		CCAGGAAAGCAGGTGGGAATGAGCGATGTTTGTTACCCAGATACTCTGATTGGGAATATTCCCAATGTCTATTACTATGCAGCCAACAACCCA<br>TCTGAAGCCACCATAGCAAAACGTCGAAGCTATGCCAATACAATCAGCTATTTGACTCCCCCAGCCGAAAATGCTGGGCTTTACAAGGGACT<br>CAAGCAGCTAAGTGAGCTGATATCATCATACCAATCGCTCAAAGACACAGGCCGTGGGCAACAGATTGTGAGCTCTATAATCAGCACTGCCA<br>AACAATGCAATCTCGACAAGGATGTGGATCTTCCTGATGAAAGTGAGGAAATCTCAGCCAAAGATCGTGACCTTGTGGTTGGGAAGGTTTAT<br>TCCAAAATCATGGAGATTGAATCTAGGCTTCTTCCTTGTGGGCTCCATATCATTGGTGAGCCTCCATCAGCCATGGAAGCAGTGGCAACACTG<br>GTTAACATTGCTGCACTAGACCGCCCCGAAGAAGGGATTTCCTCTCTCCCATCAATACTAGCCGAGACGGTTGGAAGAGGTATAGAGGAAGT<br>TTACAAAGGGAGCAACGCGGGCATCTTAAAAGATGTTGAATTACTTCGTCAAATTACTGAGGCGTCACGTGGAGCCATTTCTGCATTTGTAG<br>AGAAAACCACAAACAAGAAGGGTCAAGTGGTTGATGTAGCTGACAAACTGAGCTCAATCCTTGGTTTTGGCGTGAATGAACCGTGGGTTC<br>AGTATTTGTCGAACACTAAATTTTACAGGACTGATCGGGAGAAACTTAGGATTTTGTTTGCATTCTTAGGGGATTGCTTGAAGCTTATCGTGG<br>CAGATAATGAGTTGGGAAGTTTAAAACAAGCTTTGGAAGGAAAATACGTGGAGCCAGGTCCCGGTGGGGATCCTATTAGAAATCCAAAAGT<br>GTTGCCAACCGGGAAGAATATTCACGCACTCGATCCACAAGCTATTCCAACAACAGCAGCAATGCAGAGTGCAAAGGTTGTGGTGGAGAG<br>GTTGCTTGAGAGACAGAAGGCTGATAATGGAGGAAAGTATCCCGAGACAGTTGCACTTGTACTATGGGGAACTGATAATATCAAGACTTACG<br>GTGAGTCACTAGCTCAAGTTTTATGGATGATTGGTGTACTTCCCATTGCTGATACCTTTGGTAGAGTTAACCGAGTGGAACCAGTAAGCCTCG<br>AAGAGCTTGGCAGGCCAAGGATTGATGTTGTTGTTAATTGCTCTGGTGTATTCAGAGACCTTTTCATCAATCAGATGAATCTCCTTGACCGGG<br>CAGTGAAGATGGTAGCTGAACTAGACGAGCCAGAAGATCAAAATTACGTGAGAAAACATGCAATAGAACAAGCGAAAACCCTTGGTGTTG<br>AGGTTAGAGAAGCTGCCACAAGGGTCTTCTCCAATGCCTCTGGCTCATACTCCTCTAACATAAACCTCGCGATTGAGAATTCCTCGTGGAAC<br>GATGAGAAGCAACTTCAAGACATGTATTTGAGCCGCAAGTCCTTTGCATTCGACTGTGATGCTCCTGGTGTTGGCATGACTGAGAAACGTAA<br>AGTTTTTGAGATGGCTCTAAGTACAGCTGACGCCACATTCCAAAACCTCGACTCATCAGAAATTTCACTCACAGATGTGAGTCACTACTTTG<br>ATTCAGACCCAACCAACCTTGTACAGAACCTAAGGAAGGATGGTAAAAAGCCTAGTGCATACATTGCTGACACCACCACAGCTAATGCACA<br>GGTGCGCACGCTTTCAGAAACTGTGCGGCTTGATGCACGTACGAAGTTGTTGAATCCTAGGTGGTATGAGGGAATGTTGTCTACCGGTTATG<br>AGGGTGTGCGCGAAATTGAGAAGCGATTGACTAACACAGTTGGGTGGAGTGCAACCTCTGGTCAAGTTGACAATTGGGTGTATGAAGAGG<br>CTAACACAACTTTCATTCAAGACGAGGAAATGTTGAACAAGCTCATGAAAACCAACCCTAACTCTTTCAGAAAGTTGGTTCAGACGTTCTT<br>GGAGGCCAACGGACGTGGGTATTGGGAGACATCAGAGGACAATATCGAGAAGTTGAGGCAATTGTACTCGGAAGTTGAAGACAAGATTGA<br>AGGGATTGATCGTTAG
CsDVR	CSS0008645	ATGTCCATTTGTGTACCCTTCCATGGATTCACACTCTACTCACCAAAGGGCCAAAGCTTCAAGAGACTCTTTTCTTCTCATTTCATCAATCATG

		CTCAGGTAATCCCATTCCCATGTTCTCTACCTTTATCTCCTCTGAATTTATCAGAACCCTTTAGGTTTAGCAGAGAAAAAGCACTCAAACCCAT<br>CAGAGCTTCAATTACCCCAACTGTTGAAACCCCCAAAACTTCATTCAGAGACAAAACCCCAAAAGAAATCAATGTTTTGGTAGTGGGTTCA<br>ACTGGGTATATTGGGAAATTTGTTGCTAAAGAGTTAATTAAGAGAGGGTTTAATGTTATTGCAATTGCTAGGGAGAGTAGTGGCATTAAGGGC<br>AGAAATAGCAAAGAAGAAACCCTAGAACAGTTGAATGGGGCAAATGTTTGCTTTTCGGATGTGACCCATTTGGATTCTTTGGAGAAAAGTT<br>TGGGGAATTTAGGGGTTTCAATTGATGTTGTTGTGTCATGCCTTGCTAGCCGAAGTGGTGGTGTCAAGGACTCATGGAAGATTGATTATGAG<br>GCAACAAAGAATACTCTTGTTGCCGGTAGGAATCGTGGGGCTTCACATTTTGTTTTGTTGTCTGCAATTTGTGTGCAAAAGCCCCTTCTTGAA<br>TTTCAGCGTGCAAAGTTGAAATTCGAGGCTGAATTGATCAAACAAGCCGAAGAGGATGATGGATTTACTTATAGTATTGTGAGGCCAACGGC<br>ATTTTTCAAGAGTTTGGGTGGACAGGTTGAATTGGTGAAAGATGGGAAGCCTTATGTGATGTTTGGGGATGGGAAGTTGTGTGCTTGCAAAC<br>CTATTAGCGAGTCGGATTTGGCTTCATTTATCGCAGACTGTGTGTTGAGTCAGGATAAGTTTAATCGGGTTTTGCCCATTGGTGGACCGGGGA<br>AGGCATTGACACCATTGGAGCAAGGGGAGATTCTGTTTAGAATTTTAGGGAGAGAACCAAAATTCTTCAAAGTGCCAATTGAAATAATGGAT<br>TTTGCTATTGGGGTTCTTGATTTTCTTGTTAAGATTTTCCCTGCTATGGAAGATGCTGCTGAGTTTGGTAAAATCGGAAGGTATTATGCTGCGG<br>AGAGTATGCTGATTTTGGATCCTGAGACTGGAGAGTACAGCGCTGAGAAAACGCCAAGTTATGGGAAGGACACATTGGAAGAGTTCTTTGA<br>GAGGGTACTCAGGGAAGGAATGGCTGGTCAGGAATTAGGGGAGCAAACAATCTTCTAA
CsCLH	CSS0004684	ATGGCACTCTTAGTAGAAAGTCCAGTTGTTGTGGGAACAACAAATGTATTTGAGGAGGGAAAGTTGAGTGTGGAAAGCATAACAGTGAATG<br>AATCATCATCATCATCACCACCTAAACCATTGTTAATTGTTACACCAACGTTGGAGGGCACATATCCTGTGCTGTTGCTTTTCCATGGATTCTT<br>GATTCAGAATCAATCCTACGAACAGATTCTCCAGCATATATCTTCCCATGGATTTATAGTCGTTGCTCCTCAGTTGTACACCTTCATTTCTTCTG<br>GGACTGAGGAAGTTGATTCTGCTGCAGAAGTCACAAACTGGTTATCCGCAGGCCTTCAATCCACACTGCCTAAAAATGTTCAAGCAAATCT<br>CCTCAAACTAGCCCTTTCCGGCCATAGCCGAGGTGGAAAAGCCGCATTTGCCCTTGCACTAGGCCATGCCGAAACCCAACCAACTCTAAAA<br>TTTTCGGCTTTAATAGGGCTTGACCCCGTTGCAGGTTCAAGCATAAAAGACCAAATCGACCCCAAAATTCTCACTTTCGTCCCTCGTTCTTTC<br>GATTTATCAATCCCCATCGCCGTGATTGGCACCGGGAAGTCACCGGCGTGTGCCCCGGATGGAGTGAACCATTGTGAGTTCTTCACGGAATC<br>CAAACCACCATGTTACTATTTTTATGCCAAGGACTATGGCCATGTGGACATGTTGGATGATCCAAATATTATTTTACGTCTTGCTTGTTCGGGT<br>GGGAAGGGCGATATGGACCCATTTAAGAAATGTGTGGGAGGGATTTTTGTGGCATTTTTAAAGGCTTGTTTGGATGGTGAGAATGAAGATCT<br>GAAATCGATTGGGGATGAACCTGGTATTGCTCCCGTTACACTTGATCCGGTTATCTACATTGAAGCATAA
CsPAO	CSS0042634	ATGGCAGTAGCAAGCCCGGCGTTGGCTACAAATGTTTTTGAAGGGGGAAACTTGAGTGTGAAATCTATGAAAGTGGAGAAATCAAATCAAT

	CATCACCACCACCAAAGCCATTGTTAATTGTTACGCCAAAGTTGGAAGGCACATATCCGGTGCTGCTCTTTTTCCATGGGTTCTTGATCCACA<br>ATACCTCCTACGAAAAGCTTCTCCAACATATATCTTCCCATGGATTCATAGTCGTTGCTCCTCAGTCAATTTGGCTGGAGAAGTCACAAACTG<br>GTTGGTTAGACACTGGCCTTCAATCCATGCTCCCGAAAAATGTCAAAGCAAATCTTCACAAGCTAGCCGTTGCCGGCCATAGTAGAGGTGGA<br>AAGACTGCATTTGCCCTTGCACTAGGCCATGCCAAAACCCAAACATCCCTCAAATTTTCAGCTTTAATAGGACTTGACCCTGTTGCCGGTCG<br>AAGCGTAGATGACCAAATCGAACCCAAAATCCTCACCTATGTCCCCCGTTCTTTCGATTTTCCAGAACCGATCCCAGTTGCTATCATCGGTAC<br>TGGCTTAGGTCATGAAAAAAGTAGTGCCATATTCCCAGCATGTGCCCCAAATGGAGTGAATCATTGTGAGTTCTTCACAGAATGTAAACCAC<br>AGTGTTGGTATTTTTATGCCAACGACTATGGTCATATGGACATGTTAGATGATCCAAAATTGATGTTAAGACATGTTTTTGTGAGTGGGGAGG<br>GTGATAAGGACCTGTTTAAGAAATATTTAGGAGGGGTATTTGTAGCATTTTTAAGGGCTTGTTTGGATGATGCGAATGAAGATCTCAAGTGCA<br>TTGTGAAAGAACCTACTATTGCTCCTGTAACACAGTCATCCACATTGAAGCATAAGGTCATGTCCAAGCTCTAA
